# Management of the Two-Week Wait Pathway for Skin Cancer Patients, before and during the Pandemic: Is Virtual Consultation an Option?

**DOI:** 10.3390/jpm12081258

**Published:** 2022-07-30

**Authors:** Maria-Roxana Chiru, Sandip Hindocha, Ekaterina Burova, Gabriel-Cristian Bejan, Laura-Maria Manea, Liviu-Nicolae Ghilencea

**Affiliations:** 1 Department of Plastic Surgery, Bedford Hospital NHS Foundation Trust, Bedford MK42 9DJ, UK; sandip.hindocha@bedfordhospital.nhs.uk; 2 Department of Dermatology, Bedford Hospital NHS Foundation Trust, Bedford MK42 9DJ, UK; katya.burova@bedfordhospital.nhs.uk; 3 Department 5 of Internal Medicine, Carol Davila University of Medicine and Pharmacy, 020022 Bucharest, Romania; cristian.bejan@umfcd.ro; 4 Department of Internal Medicine and Cardiology, Coltea University Hospital, 030171 Bucharest, Romania; manea.laura.maria@gmail.com

**Keywords:** telemedicine, video consultation, skin cancer, squamous cell carcinoma, basal cell carcinoma, malignant melanoma, two-week wait pathway

## Abstract

Background: Although telemedicine emerged more than 100 years ago, the recent pandemic underlined the role of remote assessment of different diseases. The diagnoses of cutaneous conditions, especially malignant lesions, have placed significant stress on the fast-track pathway for general practitioners (GPs), dermatologists, and plastic surgeons. The aim of the study was to compare (pre- and during the pandemic) the ability of professionals to face the challenge. Methods: The study was composed of 1943 consecutive patients (mean age 61.9 ± 18.3, 53.8% female) assessed by GPs, face-to-face (988 patients, 50.8%, between October 2019 and March 2020) and by virtual (video/photo) visits (955 patients, 49.2%, between March 2020 and October 2020) for skin lesions, and referred to secondary care via the two-week wait pathway for suspected skin malignancy. Results: The two groups had similar primary skin malignancies identification rates (24.3% vs. 22.1%, *p* = 0.25). The virtual visits identified squamous cell carcinoma (SCC) better than face-to-face consultations (*p* = 0.04), but identified basal cell carcinoma less-well (BCC, *p* = 0.02), whereas malignant melanoma (MM) was equally identified in the two groups (*p* = 0.13). There was no difference in the median breach time (days) of the two-week wait pathway (12, IQR = 6 vs. 12, IQR = 5, *p* = 0.16) in the two groups. Virtual assessments (by GPs) of skin lesions suspected of malignancy, and referred via the two-week wait pathway, increased the probability of diagnosing SCC by 42.9% (*p* = 0.03), while for malignant melanomas, face-to-face and virtual consultations were alike (*p* = 0.12). Conclusions: The equivalent outcomes in the management of skin cancers (SCC, MM) via the two-week pathway through virtual consultations and face-to-face appointments underline the role of telemedicine as a reliable alternative to face-to-face assessments.

## 1. Introduction

Early diagnosis of malignant skin lesions in primary care and timely patient referral to a specialist are the cornerstones of successful and effective skin cancer pathway management. Malignant melanoma is one of the most life-threatening types of cutaneous cancers; however, early diagnoses of skin tumors are tantamount to the 20-year survival rate of nearly 100% [1]. Nonmelanoma skin cancers, such as basal cell carcinoma (BCC) and squamous cell carcinoma (SCC), though significantly less dangerous, are still critical burdens for patients and the medical system, especially when the diagnosis is overdue [2].

The NICE guidelines clearly define the situations that demand suspected skin cancer pathway referrals (i.e., an appointment with a specialist within two weeks), such as pigmented lesions that meet certain features suggestive of melanoma (with the use of a weighted seven-point checklist), lesions with dermoscopic images that increase the suspicion of melanoma, lesions with clinical aspects that resemble nodular melanoma, SCC, or BCC with concerning features [3,4]. As the referrals strictly involve visual clinical decisions, the following (justified) question naturally arises: could telediagnosis be a helpful and safe alternative in this setting?

Telemedicine use in dermatology is not a new concept, dating back to 1995, at the beginning of the internet era [5,6]. With new technological advancements in recent decades, the reliability of telemedicine has become more tangible, while the recent SARS-CoV-2 pandemic gave teledermatology—as well as telemedicine in general—well-needed boosts. Dermatology guidelines have been rapidly readjusted for global pandemic settings, to include telediagnosis as an option for patients and primary care physicians [7,8]. Most studies, even before the pandemic, both in and outside the UK, showed comparable diagnostic accuracies between face-to-face and telediagnosis options. Nevertheless, the decision-making validation through teledermatology requires evidence based on larger cohorts.

The modern world has witnessed an increasing number of skin cancers with high morbidity and mortality globally, while the most frequent types of cancer in the UK comprise basal cell carcinoma, squamous cell carcinoma, and melanoma, according to their prevalence [7]. Early diagnosis of skin cancer is critically important, as it may prevent complications and death when managed properly. Many protocols around the world, including from the UK, set patients on fast-track pathways to secondary care, i.e., patients are seen by dermatologists and/or plastic surgeons within two weeks from a referral (a two-week wait pathway).

In the last two years, the world has seen the spread of COVID-19, a highly-contagious disease. Many facilities, including hospitals and general practitioner facilities, had to adapt their protocols in order to provide the best healthcare services to skin cancer patients. New and old communication systems, including video clinics, have been used by GPs to assess patients prior to referring them to specialists, to avoid putting these patients at risk for SARS-CoV-2 infection.

In the present study, we compared the diagnostic accuracy in primary care via the fast-track pathway for suspicious cutaneous lesions between face-to-face assessments (before the pandemic) and virtual (video/photo) consultations (during the pandemic).

## 2. Materials and Methods

### 2.1. Study Design

This was a registry retrospective study involving 2037 consecutive patients with suspected skin cancer. The patients’ general practitioners (GPs) referred them to Bedford Hospital between October 2019 and October 2020 under the two-week wait pathway rule. Patients with suspected skin cancer were registered and seen in the specialist clinics for diagnosis and treatment. The study covered six months during the COVID-19 pandemic (2020) when the patients were seen via virtual video consultations by the GPs, and six months prior (2019) when the assessment was done exclusively face-to-face. The patients were initially assessed by general practitioners (GPs); lesions suspected of malignancy were referred to specialists (dermatologists and plastic surgeons) via outpatient assessment (OPA) joint clinics for further evaluation and treatment. Histopathology analyses were performed on any skin lesions. Demographic, clinical, and histopathological data were collected and used for further analyses.

### 2.2. Inclusion Criteria

The patients had to meet the following inclusion criteria to be eligible for the study: referral from a general practitioner via the two-week wait rule and presenting a skin lesion suspected of malignancy as the main reason for the hospital presentation. The GP completed a referral form with the characteristics of the suspected lesions.

### 2.3. Exclusion Criteria

**Figure 1:**
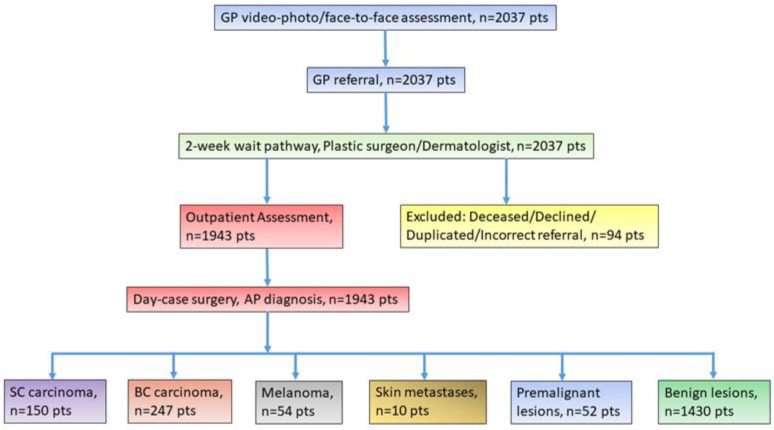
Flowchart of the study. GP, general practitioner; AP, anatomopathology; SC, squamous cell; BC, basal cell.

The exclusion criteria applied to patients who declined the OPA appointment as a result of COVID-19, opted for private care, were deceased prior to the OPA, or were incorrectly referred or unfit for assessment/treatment due to COVID-19 (Figure 1). All patients included in the study gave their written informed consent, which is in line with the principles outlined in the Declaration of Helsinki.

### 2.4. Statistical Analysis

Data for continuous variables are presented as mean ± SE (%) when the distribution is uniform and as the median and interquartile range (IQR) for non-Gaussian distribution.

We performed comparisons of the central tendencies of the baseline characteristics and endpoints of the two groups (face-to-face and virtual consultations) using a Student’s t-test for normally distributed continuous variables and nonparametric tests (Mann–Whitney U rank-sum test) to compare the non-normally distributed continuous variables. Categorical data are reported as numbers (percentages %), with group comparisons using Pearson’s chi-square test and Fischer’s exact test. A positive predictive value (PPV) was calculated for each type of cancer in both groups.

We measured the strength of the association between the diagnosis of SCC/melanoma/BCC and the type of assessment (virtual versus face-to-face); moreover, a forest plot was constructed.

All *p*-values were two-sided and a *p*-value < 0.05 was considered statistically significant. The statistical analysis was performed with the Statistical Package for the Social Sciences (SPSS) program, version 21 software (IBM SPSS Statistics, Armonk, NY, USA; IBM Corp.).

## 3. Results

### 3.1. Patient Population

The study population consisted of 2037 patients with suspected skin cancer referred to the joint outpatient clinics of Dermatology and Plastic Surgery at Bedford Hospital between October 2019 and October 2020 for specialist assessments and treatment. After applying the exclusion criteria, 1943 patients were assessed (Figure 1).

### 3.2. Baseline Characteristics

The mean age of the patients was 61.9 ± 18.3 years; patients were predominantly female (53.8%). The cohort was characterized by a moderate rate of squamous cell carcinoma (SCC, *n* = 150, 7.7%) and basal cell carcinoma (BCC, *n* = 247, 12.7%); 54 patients (2.8%) had malignant melanoma and melanoma in situ (MM). The total primary skin malignant conditions were responsible for 451 cases (23.2%), while metastases and other secondary skin cancers were responsible for 0.5% (10 cases). Premalignant lesions (Bowen’s disease and lentigo maligna) were composed of 52 cases (2.7%).

Patients were also diagnosed with benign conditions (*n* = 1430, 73.6%), such as seborrheic keratosis (SK, *n* = 444, 22.85%), actinic keratosis (AK, *n* = 131, 6.74%), dysplastic, intradermal or compound naevus (*n* = 260, 13.38%), and other benign nonspecific lesions (papilloma, verruca, insect bite, skin tag, histiocytoma, trichofolliculoma, trichilemmoma, lipoma, idiopathic hypomelanosis, acne, freckle, neuroma, xanthoma, cellular neurothekeoma, pilomatrixoma, pityriasis rubra, *n* = 82, 4.22%). A very small number of patients declined OPA (*n* = 63, 3.1%) or opted for private care (2.2%). A total of 6 patients were deceased prior to the OPA (0.3%), while 12 patients had duplicate referrals (0.6%). Twenty-seven patients had no lesions at the OPA (1.3%) (Table 1). The conditions identified in the anatomopathology assessment are listed in Table 1.

The number of patients who had virtual video/photo assessments is similar to the number of patients who had face-to-face assessments before the pandemic (49.2% vs. 50.8%) (Table 2). The two groups of patients—who had face-to-face consultations and virtual video/photo consultations—were similar in terms of age (62.5 ± 18.0, 95%CI = 61.4–63.7 vs. 61.2 ± 18.7, 95%CI = 60.0–61.5, *p* = 0.097), gender (female: *n* = 535, 54.1% vs. *n* = 510, 53.4%, *p* = 0.75), and the median time to first being seen (12, IQR = 5 vs. 12, IQR = 6, *p* = 0.16). The 14-day breach time was slightly (but not statistically) higher in the virtual video/photo group (*n* = 87; 8.7% vs. *n* = 106; 11.1%, *p* = 0.081). The two groups showed no differences in the frequencies of diagnoses concerning the main dermatological conditions, apart from the two major cell carcinomas (squamous and basal) (*p* = 0.04 and *p* = 0.02, respectively), but not in malignant melanoma/melanoma in situ (*p* = 0.131). The number of patients who declined OPA (due to COVID-19, declined treatment, patient was away, did not attend the treatment, or opted for private care) was similar in the two groups (*n* = 34; 3.3% vs. *n* = 29; 2.9%, *p* = 0.61), with incorrect referral significantly higher in the virtual video group (*n* = 2; 0.2% vs. *n* = 9; 0.9%, *p* = 0.036).

All cases were treated locally and there was no patient upgraded from the secondary to the tertiary center. No patient died after the excision procedure before or during the pandemic. The 14-day breach time was statistically non-significantly higher in virtual consultations (10.6% vs. 9.6%), but this was explicable as the pandemic raised availability questions regarding the already crowded OPAs.

The positive predictive value for the correct referral of skin cancer (in the video consultation versus face-to-face approach for SCC, MM, and BCC) was 9% vs. 6.5%, *p* = 0.037; 2.2% vs. 3.3%, *p* = 0.126; and 10.9% vs. 14.5%, *p* = 0.018, respectively. The positive predictive value for the appropriate referral of skin cancer (regardless of the type of primary malignancy) was 22.09% (211/955) for video consultations vs. 24.29% (240/988) for face-to-face consultations (*p* = 0.25).

**Figure 2:**
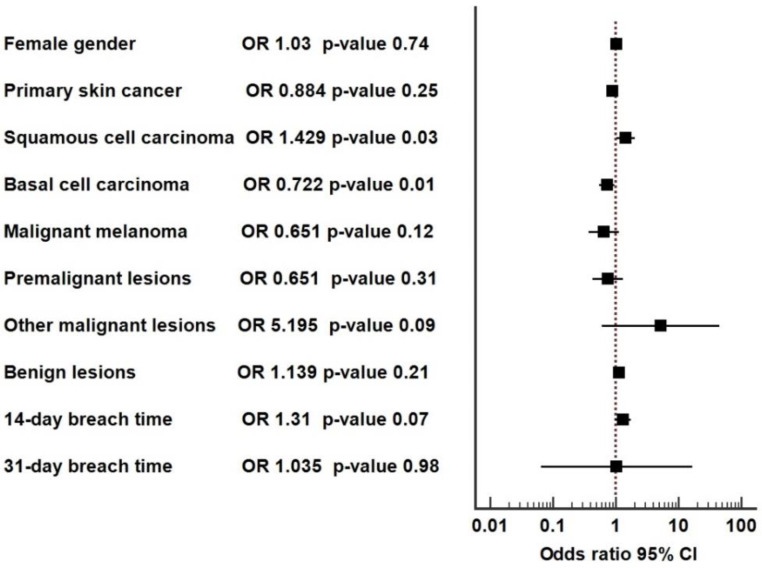
Forest plot for the face-to-face (**left**) and virtual video/photo consultation (**right**).

The odds ratio (OR) of SCC in the virtual versus face-to-face consultation groups was 1.429 (95%CI = 1.020−2.0); the SCC was more frequent in the virtual assessment with 42.9% (Figure 2). One explanation would be that virtual consultations during the lockdown were more affordable than face-to-face assessments from before the pandemic when patients had difficulties in finding slots for consultations with their GPs. The ORs of malignant melanoma and premalignant lesions in the virtual versus face-to-face assessments were both subunits (0.651, *p* = 0.12 and 0.651, *p* = 0.31, respectively), but there was no statistically significant difference between the two types of consultations. The OR of basal cell carcinoma for the two groups was 0.722 (95%CI = 0.551–0.946, *p* = 0.01); the virtual consultations identified 27.8% fewer cases of BCC compared with face-to-face consultations. The two-week wait pathway was not dedicated to basal cell carcinoma, as it was referred via the urgent pathway due to the low degree of metastasis. The 14-day breach time, although more frequent in the virtual assessments, was not statistically significant when compared to face-to-face consultations (OR = 1.31, 95%CI = 0.971–1.767, *p* = 0.07).

## 4. Discussion

### 4.1. Rationale for the Study

The recent SARS-CoV-2 pandemic enabled an unexpected push toward telemedicine at the beginning of 2020, which included almost all clinical medical specialties, including dermatology. One of the most telling applications of teledermatology involves rapid management pathways for skin cancer referrals, solely based on remote consultations. Nonetheless, one important issue raised, especially during the pandemic, involved the accuracy of diagnoses. The pandemic, though unfortunate in many aspects, may be a source of critical information regarding the precision of telediagnosis in dermatology. As data continue to be derived retrospectively, the lessons learned may be applied in the post-pandemic medical system.

### 4.2. Added Value to the Literature

Our study’s strongest point was the large number of patients included, which gave weight to the statistical results. Moreover, the gold standard for diagnosis involves histopathological results; however, for most studies that tackled the same issue, the interobserver variability was reduced, as the examining doctors were the same both before and after the beginning of the pandemic. The originality of this study lies in the slightly different approach that we took: we did not assess the clinical diagnostic accuracy of specific types of skin cancer, instead, we evaluated the overall pick-up rate for skin neoplasia. To the best of our knowledge, our work is the single study that compares (between telediagnosis and face-to-face diagnosis) the positive predictive values of a GP’s two-week pathway referral.

### 4.3. Comparison with Other Studies

Telemedicine was a rapidly evolving field even before the pandemic; advancements in technology have been followed closely throughout the years [9]. The lockdowns due to the recent SARS-CoV-2 outbreak forced physicians and patients alike to find solutions to certain medical problems through telemedicine (to prevent patients from being unnecessarily exposed) [10]. Undeniably, dermatology is one of the most suitable specialties for telemedicine; in essence, teledermatology involves remote consultations given by general practitioners or skin specialists, using varied modalities, such as video consultations, stores, or forward images, for screening, diagnostic purposes, or therapeutically adjustments [8]. The benefits of teledermatology are plentiful: increased accessibility for patients who live in remote areas or atypical environments (e.g., by sea, in the army) [11,12], reduced wait times for patients, diminishing the number of unnecessary referrals [13], increasing the cost-efficiency of medical acts [14], ensuring continuity in the monitoring process. The most crucial part of teledermatology involves the diagnosis of skin neoplasm.

There are several comprehensive, qualitative, and recent meta-analyses dedicated to the telediagnosis of skin cancer [15,16,17]; nevertheless, the vast majority of studies have enrolled less than 100 patients, with few surpassing 1000 patients. Moreover, most of them were conducted before the pandemic, when telemedicine was not a central part of medical systems. Finnane et al., after analyzing 21 studies, concluded that telediagnosis has lower diagnostic accuracy than classic diagnosis but these results are open to interpretation, provided that there are some serious and heterogeneous methodological limitations among the analyzed studies. The authors also underlined the need for confirmation of these results from larger studies [17]. Our study addresses some of these limitations: the number of patients was significantly higher than any of the studies included, the reference standard was exclusively the histopathological exam, and the variation in interobserver reliability was decreased, as the study included patients referred by the same doctors before and during the pandemic.

Another rigorous pre-pandemic meta-analysis concluded that the telediagnosis of skin cancer is a good alternative to face-to-face diagnosis, but reliable evidence is lacking due to the great heterogeneities of the assessed studies, most of which were made in secondary care settings, contrary to our work [16]. The authors suggest that a solution for an appropriate triage of the lesions could be a “more widely” defined threshold to identify malignancy and they underlined that the crucial part of teledermatology is not the precise classification of the lesions, but the decision on whether the patient should be referred for a face-to-face consult. Somewhat in agreement with this view, when we analyzed the referral precision of the primary care physicians, we considered malignant lesions as a unique category. Regardless of the definition of “malignant lesions” (with or without premalignant lesions and metastases), the PPV determined in the two cohorts varied between 22.9% and 27.42%. Most of the studies carried out in the UK found lower pick-up rates for malignancies (whose definitions were not homogeneous), varying between 10% and 34.5% [18,19,20,21], including a 13.2% rate in a recent study [4]. As to whether the accuracy of the two-week referral pathway was too weak is highly debatable; a low suspicion threshold for malignancy, especially for melanoma, is desirable, leading to an inherent increase in the number of false positive cases.

Most importantly, the PPV for video consultation vs. face-to-face consultation was significantly higher in the SCC diagnosis (*p* = 0.037), similar for MM (*p* = 0.126), and significantly lower for BCC at risk (*p* = 0.018), but the two methods were not statistically different in our study (*p* = 0.25), implying that they are equally performant in identifying skin cancer overall. Furthermore, there were better assessments of SCC with virtual video consultations than with face-to-face consultations (86/955 vs. 64/988, OR = 1.429, *p* = 0.04). A possible explanation for this discrepancy could be a selective increase in the addressability of patients with SCC, as a virtual appointment is more convenient and far less time-consuming than a classic consult. Contrarily, there were fewer BCCs referred through video consultations than face-to-face consultations (104/955 vs. 143/988, OR = 0.722, *p* = 0.02), which could have translated to better selections during the pandemic of the BCC cases that needed rapid referrals to specialists, according to NICE guidelines: “skin lesion that raises the suspicion of a basal cell carcinoma if there is particular concern that a delay may have a significant impact, because of factors such as lesion site or size” [3]. An additional explanation is based on the fact that there was a lower number of suspected basal cell carcinoma referrals (by family doctors during the pandemic) via the two-week wait pathway, as there was a specific dedicated 30-day urgent pathway for skin cancers (including basal cell carcinoma) other than squamous cell carcinoma and malignant melanoma. Nevertheless, during the pandemic, due to the restrictions, including in the healthcare sector, family doctors had stricter understandings of the guidelines and limited the two-week wait pathways to referrals related to squamous cell carcinoma, malignant melanoma, and basal cell carcinoma, with increased concerns, and made use of the urgent pathway for basal cell carcinoma, instead. The assessment of MM, as well as that of premalignant lesions (Bowen’s disease, lentigo malignant), with a video consultation was similar with a face-to-face consultation (OR = 0.651, *p* = 0.13 and OR = 0.753, *p* = 0.32, respectively). Of note, the suitability of teledermatology as a triage tool for malignant melanoma was highlighted by Finnane et al., showing that all melanoma cases were referred to as high priority in certain studies included in the meta-analysis [17].

As a result, the referral via the two-week wait pathway of skin lesions suspected of malignancy, after the virtual assessment by the GP, improved the likelihood of diagnosing SCC by 42.9% (*p* = 0.03), while for the malignant melanomas, the face-to-face and virtual consultations were equivalent (*p* = 0.12).

In our cohort, only 9.9% of the patients had to wait more than two weeks for a dermatology consult, which is a significantly lower value than the one reported by Redai and O’Connor in a study undertaken in the Queen’s Hospital catchment area, in 2019, on 102 referred patients (35%) [4]. However, data derived from the Northern Cancer Network in the first wave of the SARS-CoV-2 pandemic showed that the median wait time was significantly reduced compared with the same time frame in 2019 [22]. For our two cohorts of patients, there were no significant differences in the wait times (days) for the dermatology consults (12, IQR = 5 vs. 12, IQR = 6).

These are the reasons why we believe that a high-quality photo of the suspected lesion of SCC would improve the breach time. A high-resolution photo accompanying the referral would improve the hospital triage system (regarding suspected SCC/melanoma patients) by upgrading the cases sent via routine/urgent routes, whereas the referral of the suspected SCC in the two-week wait pathway may be given the right priority after the outpatient clinic assessment by the dermatologist/plastic surgeon.

### 4.4. Limitations of the Study

The retrospective nature of the study includes its inherent limitations; the study did not include patients whose lesions were deemed benign by the GP, rendering impossible the determining of sensitivity, sensibility, or the negative predictive value of skin cancer telediagnosis. In addition, dermoscopic techniques were not included and the primary care physician’s level of knowledge regarding skin neoplasia was not assessed.

## 5. Conclusions

Virtual assessments (by GPs) of skin lesions suspected of malignancy and referrals via the two-week wait pathway increase the probability of diagnosing SCC, while for MM, face-to-face and virtual consultations are alike. The comparable outcomes in the management of skin cancers (SCC, MM) via the two-week wait pathway (through virtual consultations and face-to-face appointments) highlight the role of telemedicine as a reliable alternative to face-to-face assessments.

## Figures and Tables

**Table 1:** Key baseline characteristics of the two-week wait pathway patients (*n* = 1943).

Characteristics	Value
Number (%)	1943 (100%)
Age at diagnosis, yo, mean ± SD (95% CI)	61.9 ± 18.3
Female gender, *n* (%)	1045 (53.8%)
Time to first being seen (days), median (IQR)	12 (6)
Total skin malignant/premalignant lesions, *n* (%)	513 (26.4%)
Skin primary malignancies (SCC, MM, BCC,), *n* (%)	451 (23.2%)
SCC, *n* (%)	150 (7.7%)
MM, *n* (%)	54 (2.8%)
BCC, *n* (%)	247 (12.7%)
Metastases and other secondary malignancies, *n* (%)	10 (0.5%)
Premalignant conditions (Bowen’s disease/lentigo maligna), *n* (%)	52 (2.7%)
Skin benign lesions, *n* (%)	1430 (73.6%)
14-day breach time, *n* (%)	192 (9.9%)
31-day breach time, *n* (%)	2 (0.1%)
62-day breach time, *n* (%)	1 (0.05%)
No lesion present at the OPA, *n* (%)	27/1943 (1.3%)
Declined the OPA, *n* (%)	63/2037 (3.1%)
Deceased prior to the OPA, *n* (%)	6/2037 (0.3%)
Duplicate referrals, *n* (%)	12/2037 (0.6%)
Incorrect referrals, *n* (%)	11/2037 (0.5%)
Unfit for the OPA, *n* (%)	2/2037 (0.1%)

SCC, squamous cell carcinoma; MM, malignant melanoma and melanoma in situ; BCC, basal cell carcinoma; OPA, outpatient assessment.

**Table 2:** Comparison of the two-week wait pathway before and during the pandemic groups.

Characteristic	Face-to-Face-Consultation	Virtual Video Consultation	*p*-Value
Number (%)	988 (50.8%)	955 (49.2%)	
Age, years, mean ± SD (95% CI)	62.5 ± 18.0 (61.4–63.7)	61.2 ± 18.7 (60.0–61.5)	0.09
Female gender, *n* (%)	535 (54.1%)	510 (53.4%)	0.75
Time to first being seen (days), median (IQR)	12 (5)	12 (6)	0.25
Primary skin malignancy, *n* (%)	240 (24.3%)	211 (22.1%)	0.25
SCC, *n* (%)	64 (6.5%)	86 (9%)	0.04
MM, *n* (%)	33 (3.3%)	21 (2.2%)	0.13
BCC, *n* (%)	143 (14.5%)	104 (10.9%)	0.02
Metastases/other malignant lesions, *n* (%)	3 (0.3%)	7 (0.7%)	0.21
Premalignant lesions, *n* (%)	30 (3%)	22 (2.3%)	0.32
Benign lesions, *n* (%)	715 (72.4%)	715 (74.9%)	0.21
14-day breach time, *n* (%)	86 (8.7%)	106 (11.1%)	0.08
31-day breach time, *n* (%)	1 (0.1%)	1 (0.1%)	1
62-day breach time, *n* (%)	1 (0.1%)	0 (0%)	1
Excluded from the study, *n* (%)	43/2037 (4.2%)	51/2037 (5.1%)	0.34
Deceased prior to the OPA, *n* (%)	3/1031 (0.3%)	3/1006 (0.3%)	1
Declined the OPA, *n* (%)	34/1031 (3.3%)	29/1006 (2.9%)	0.61
Incorrect referral, *n* (%)	2/1031 (0.2%)	9/1006 (0.9%)	0.03
Duplication, *n* (%)	3/1031 (0.3%)	9/1006 (0.9%)	0.09
Unfit for the OPA, *n* (%)	1/1031 (0.1%)	1/1006 (0.1%)	1

## Data Availability

Data supporting the reported results can be viewed upon request (for good reason) to the delegate responsible at the hospital.
